# Reassessing pharmacologic amnesia: benzodiazepines and intraoperative awareness in adults: a perspective on the limits of the evidence and the primacy of anesthetic depth

**DOI:** 10.3389/fmed.2026.1957985

**Published:** 2026-10-27

**Authors:** Jianwei Guo, Yan Cheng, Minmin Yi

**Affiliations:** Department of Anesthesiology, Shaanxi Provincial People’s Hospital, Xi'an, Shaanxi, China

**Keywords:** anesthetic depth, benzodiazepines, intraoperative awareness, perioperative care, postoperative delirium, processed electroencephalographic monitoring

## Abstract

Perioperative benzodiazepines (BZDs) have long been given to adults in part for their anterograde amnestic effect: a pharmacologic safety net intended to prevent explicit memories of intraoperative awareness, should it occur. Recent evidence calls for a reassessment of that rationale, though not for its abandonment. In the 2025 B-Free trial (n = 19,768), a cluster-randomized crossover trial in adult cardiac surgery, no patient in either the BZD-free or the BZD-liberal group spontaneously reported awareness. Awareness is rare, however, and the trial ascertained it by spontaneous report rather than structured interview, so this observation says little about small or infrequent effects. The trial was powered for postoperative delirium. Its primary intention-to-treat analysis was neutral (14.0% vs 14.9%; adjusted odds ratio 0.92, 95% confidence interval 0.84 to 1.01; *P* = 0.07). A per-protocol analysis suggested lower odds of delirium (odds ratio 0.90, 95% confidence interval 0.82 to 0.99; *P* = 0.02), an exploratory result open to selection and adherence bias. Systematic reviews and randomized trials do not establish a reliable awareness-preventive benefit of routine BZD administration, and several suggest possible harm. We argue that prevention of intraoperative awareness should rest on the assurance of adequate anesthetic depth, supported by preoperative risk stratification, monitoring beyond vital signs, and protocolized depth management. We also set out the principal limitations of the evidence base.

## Introduction

Perioperative BZDs have been given to adults in part for their anterograde amnestic effect: a pharmacologic safety net intended to spare patients explicit memories of intraoperative awareness, should it occur. A substantial proportion of surgical patients receive a benzodiazepine perioperatively, and what counts as an acceptable perioperative drug regimen varies across institutions and eras. Epidemiological data identify preoperative sedative-hypnotic medication use, of which benzodiazepines are the most common class, as a marker of adverse postoperative outcomes in surgical populations (1). The underlying logic is easy to follow. Even if a patient momentarily regains consciousness during surgery, the amnestic properties of BZDs should prevent a lasting explicit memory from forming, and the psychological consequences of awareness should therefore be mitigated. The scope of this article is limited to that specific rationale.

The stakes are considerable. The overall incidence of intraoperative awareness with explicit recall is approximately 0.1% to 0.2% in routine noncardiac, nonobstetric surgery (2, 3), but it is higher in several at-risk populations: 0.4% to 1.5% in cardiac surgery, approximately 0.2% to 1% in general anesthesia for cesarean delivery, and up to approximately 11% to 43% in emergency trauma surgery (3–6). These estimates derive from studies of varying methodological rigor, using different populations, case mixes and methods of ascertainment (3, 4). For cesarean delivery, a recent prospective multicentre cohort study of 3,115 obstetric patients in England, in which awareness was ascertained by structured questioning repeated over 30 days, reported an incidence of 1 in 212 caesarean general anesthetics (5). In the fifth National Audit Project (NAP5) of the United Kingdom and Ireland, the incidence of patient reports of accidental awareness during general anesthesia was approximately 1 in 19,600. Female sex, younger age, obesity and the use of neuromuscular blockade were among the associated risk factors, and NAP5 also refined the terminology used in this field, which we adopt (7). The psychological sequelae of awareness can be substantial (8–10). Early estimates of posttraumatic stress disorder in 30% to 70% of affected patients came from small and older series in which questionnaire-based psychological symptoms were not always distinguished from a formal clinical diagnosis; more recent cohorts interviewed systematically report markedly lower and more variable prevalence, alongside sleep disturbance, phobic anxiety and persistent fear of anesthesia (9, 11). Intraoperative awareness remains among the most feared complications of anesthesia for patients and practitioners alike, and the appeal of a pharmacologic safeguard is easy to understand.

The evidence supporting routine BZD administration for awareness prevention is less secure than its long-standing use might suggest. Available trials and systematic reviews do not demonstrate a reliable benefit for the prevention of awareness with explicit recall. Observational data associate routine BZD administration with adverse postoperative outcomes, including delirium (12, 13), and the evidence is insufficient to exclude a modest protective effect on awareness (14). We argue here for a reassessment of how the rationale for routine BZD use is framed, shifting emphasis from pharmacologic amnesia toward the assurance of adequate anesthetic depth. We do not argue for categorical abandonment of perioperative BZDs, which retain legitimate indications outside awareness prevention and recognized roles in managing patients at high risk of awareness (15).

### The rationale for pharmacologic amnesia

The appeal of BZD-based awareness prevention rests on a well-defined pharmacology. BZDs potentiate *γ*-aminobutyric acid type A (GABA_A_) receptor-mediated inhibition and thereby suppress hippocampal circuits that support the consolidation of explicit memory, while the arousal-suppressing hypnotic effect of a given dose is less predictable (16, 17). The American Society of Anesthesiologists Practice Advisory for Intraoperative Awareness and Brain Function Monitoring includes BZD administration among the interventions to be considered in patients at increased risk of awareness, a recommendation resting on expert opinion and not on randomized evidence (15). That recommendation is not unfounded, and BZDs can produce unconsciousness when dose-adjusted as a primary anesthetic. Our concern is narrower: the adjunctive use of BZDs as a substitute for the assurance of adequate anesthetic depth. Amnesia is an unreliable clinical safeguard for three reasons. BZDs lack analgesic properties and do not suppress cortical responses to noxious stimulation (17, 18), so a patient can undergo a distressing experience without being able to recall or report it. The amnesia produced by the doses commonly given as premedication, such as midazolam 2–3 mg intravenously, is neither complete nor predictable (16, 17). And both the amnestic and the hypnotic responses to a given dose vary appreciably between patients (16). Amnesia, awareness, nociceptive processing and anesthetic depth are distinct phenomena. Evidence that a drug impairs memory formation cannot on its own establish that it prevents the conscious experience of surgery (19, 20). This is the conceptual problem we address below.

### Pharmacologic amnesia and anesthetic depth: a conceptual reassessment

An overly simple dichotomy between pharmacologic amnesia and anesthetic depth obscures the fact that the two act at different points along a causal chain. Depth determines whether a conscious experience occurs; the capacity to encode and retrieve a memory of that experience is a separate process that can dissociate from its occurrence (19, 20). Evidence of preserved memory function is therefore not interchangeable with evidence of preserved consciousness, and unreported recall is a weak surrogate for intact consciousness. The distinction matters clinically in two settings. Where adequate anesthetic depth is reliably achievable, the incremental value of drug-induced amnesia is limited, and its routine use exposes patients to adverse effects, including delirium (12, 13). Where adequate depth cannot always be assured, as during cardiac surgery, in trauma resuscitation, in obstetric general anesthesia, or in the presence of hemodynamic instability, a recognized role for BZDs in patients at high risk of awareness persists, and our argument does not extend to those circumstances (15). Neither does it set anesthetic depth and BZD administration against one another as mutually exclusive. The two mechanisms are complementary, and the clinical question is how much each contributes.

#### The evidence base and its limitations

The evidentiary foundation for BZD use in awareness prevention is weaker than its long-standing clinical acceptance suggests (Table 1). A 2023 systematic review and meta-analysis encompassing 34 randomized controlled trials (*n* = 4,354) and 9 observational studies (*n* = 3,309) reported that BZDs might reduce the risk of definite intraoperative awareness (relative risk [RR] 0.26; 95% confidence interval [CI] 0.12–0.58; *P* = 0.001), an effect its authors rated as very low certainty evidence. That qualification matters when the finding is invoked in support of guidelines (14). The included trials were predominantly small, single-center and unblinded, and 73.5% were judged to be at high risk of bias. Most were conducted in obstetric populations and are now several decades old, and only two used a structured instrument such as the Brice questionnaire to ascertain awareness. The review also reported a significant subgroup difference for the awareness outcome (*P* = 0.03) (14). The rarity of the outcome complicates the meta-analysis. The review's zero-event meta-analysis is not evidence of the absence of an effect, and we do not read it as an artifact. When the control event rate is close to zero, the continuity correction used to compute pooled risk ratios generates numerically unstable estimates, and the authors of the review interpret this analysis cautiously (14). Awareness was analysed as a single dichotomous outcome and was not combined with other endpoints. The same review found no statistically significant increase in postoperative delirium with BZDs in the randomized trials (RR 1.43; 95% CI 0.90–2.27), with the excess risk concentrated in comparisons against dexmedetomidine (RR 1.83; 95% CI 1.24–2.72) (14). A balanced reading therefore supports neither a clear awareness-preventive benefit nor a clearly established delirium signal from randomized data, and the certainty of the evidence for most outcomes remains low or very low. Our argument is one of insufficiently demonstrated benefit relative to potential harm, not of proven absence of effect. The reviews by Wang and colleagues and by Ge and colleagues draw on an overlapping evidence base, share authors with one another and with the B-Free investigators, and have prompted published editorials and methodological correspondence. They are not independent confirmations of one another. The editorial responding specifically to the Wang review (Evered and Pryor, 2023) questioned the robustness of that analysis, noting in particular the potential for bias arising from the composition of the included trials and from the handling of delirium as an outcome, and further methodological points were raised in subsequent correspondence. Each review should be read alongside those commentaries (14, 21, 23). Related evidence comes from the 2016 Cochrane review of anaesthetic interventions for awareness prevention, which also examined drug regimens. Its meta-analyses of anaesthetic drugs drew on nine small trials (1,290 participants), largely older comparisons, and reported that benzodiazepines reduced the risk of awareness compared with thiopental, ketamine or placebo; the quality of this evidence was graded low or very low, most of the included trials had unclear risk-of-bias domains, and 115 of the 160 trials in the review could not be used in the meta-analyses because they reported zero awareness events (24). Those trials studied benzodiazepines mainly as induction or maintenance agents rather than as adjunctive premedication, so they are consistent with a possible awareness-sparing effect of benzodiazepine anesthesia, but they do not demonstrate a benefit of the adjunctive amnestic use that is our focus (24).

**Table 1:** Summary of key evidence on perioperative benzodiazepine use and intraoperative awareness in adults. Methodological quality and design descriptors were assigned independently by two authors (J.G. and Y.C.) using design-based criteria (randomization, blinding, adequacy of awareness ascertainment and sample size), with discrepancies resolved by discussion; GRADE certainty is reported as rated by the original systematic reviews, and no independent GRADE assessment was performed.

Study (Year)	Design	Sample Size	Key Finding	Methodological Quality of the Study	Limitations to Interpretation
Wang 2023 (14) (Br J Anaesth)	Systematic review and meta-analysis (34 RCTs, 9 observational studies)	4,354 (RCTs); 3,309 (observational)	Possible reduction in awareness with BZDs (RR 0.26; 95% CI 0.12–0.58), rated very low certainty; no significant increase in delirium in RCTs (RR 1.43; 95% CI 0.90–2.27), with excess risk concentrated in comparisons with dexmedetomidine (RR 1.83; 95% CI 1.24–2.72)	GRADE: very low certainty for awareness	Predominantly small, single-centre, unblinded trials; 73.5% at high risk of bias; mostly obstetric and historical; only 2 trials used the Brice questionnaire; significant subgroup effect (*P* = 0.03); zero-event analysis numerically unstable
Modaberi 2026 (25) (Saudi Dent J)	Double-blind RCT	56	No significant difference in awareness (3.6% vs 10.7%; *P* = 0.611)	Single-centre RCT; small sample; randomization and blinding reported	Inconclusive: sample far too small to exclude a clinically important difference; control group awareness rate incompatible with epidemiological estimates; single dose at induction only
Spence 2025 (26) (JAMA Surg) — B-Free	Pragmatic cluster-randomized crossover trial (institutional policy)	19,768	No spontaneously reported awareness in either group; intention-to-treat neutral for delirium (14.0% vs 14.9%; aOR 0.92; 95% CI 0.84–1.01; *P* = 0.07); per-protocol aOR 0.90 (95% CI 0.82–0.99; *P* = 0.02)	High methodological quality for the trial as conducted; not powered for awareness	Awareness ascertained by spontaneous report only; zero events uninformative given the expected number of events; per-protocol result exploratory and susceptible to selection and adherence bias; conducted exclusively in adult cardiac surgery (limited external validity)
Liang 2025 (28) (BMC Anesthesiol)	Randomized controlled non-inferiority trial	100	No awareness events in either group; comparable anesthetic success; less hypotension and injection-site pain with remimazolam	Randomized, single-centre, non-inferiority design	Awareness not the primary outcome and not formally powered; single-centre day-case population; provides indirect evidence only
Song 2025 (29) (J Clin Anesth)	Systematic review and meta-analysis (19 RCTs)	3,031	No significant difference in awareness risk between remimazolam and propofol (RR 0.33; 95% CI 0.01–8.16; *P* = 0.502); less intraoperative hypotension with remimazolam (RR 0.65; 95% CI 0.46–0.92)	Low to moderate certainty (awareness was a secondary outcome)	Extremely wide confidence interval compatible with substantial benefit or harm; awareness not the primary outcome; possible higher risk of delirium with remimazolam in ASA III–IV patients noted by the authors; indirect evidence for the question at issue
Salameh 2026 (30) (J Cardiothorac Vasc Anesth)	GRADE-assessed systematic review and meta-analysis (9 studies)	903	Fewer intraoperative hypotensive events (RR 0.50; 95% CI 0.31–0.80) and faster emergence with remimazolam vs propofol in adult cardiac surgery	GRADE-assessed; certainty varied across outcomes	Addresses hemodynamic stability and recovery, not awareness prevention; indirect evidence; small total sample
Ge 2025 (12) (Br J Anaesth)	Systematic review and meta-analysis (123 RCTs; 93 in quantitative synthesis)	12,921	Perioperative BZDs did not improve patient-reported outcomes and may increase postoperative anxiety	GRADE-assessed systematic review of RCTs	Assessed patient-reported outcomes and did not evaluate delirium; shares authorship and evidence base with the other review cited, so the two are not independent

Modaberi and colleagues conducted a double-blinded randomized controlled trial in orthognathic surgery (*n* = 56) that shows how difficult it is to demonstrate this effect in small samples (25). Patients receiving midazolam 0.1 mg/kg at induction had a reported awareness rate of 3.6% (1/28) compared with 10.7% (3/28) in the placebo group, a numerical difference of 7.1 percentage points that did not reach statistical significance (*P* = 0.611). The sample is small, and the observed 10.7% rate in the control group is one to two orders of magnitude higher than the incidence of awareness with explicit recall reported in the epidemiological literature cited above (2, 3, 7). The discrepancy points either to a highly selected population or to a high false-positive rate of the assessment method. The trial is best regarded as inconclusive.

The most substantial randomized evidence to date comes from the 2025 B-Free trial, a pragmatic multicenter cluster-randomized crossover trial that compared a BZD-free with a BZD-liberal policy in adult cardiac surgery, the population traditionally considered most in need of BZDs (26). Conducted across 20 North American cardiac surgery centers (18 in Canada, 2 in the United States) and enrolling 19,768 patients, the trial alternated institutional policies in 4-week cycles. During restrictive periods no BZDs were to be used during cardiac surgery; during liberal periods a minimum midazolam-equivalent dose of 0.03 mg/kg ideal body weight was mandated. The cluster-crossover design limited the contamination that patient-level randomization would have permitted, since anesthetic practice patterns are difficult to modify at the level of the individual patient. No patient in either group spontaneously reported intraoperative awareness. This observation is largely uninformative about the effect of BZDs on awareness. Awareness was ascertained by spontaneous report, not by a systematically administered postoperative interview such as the structured Brice questionnaire, an approach that is known to underestimate its incidence (27). The expected number of events is also small even in this large sample. With the incidences cited for cardiac surgery (0.4–1.5%), approximately 80 to 300 events would have been expected among 19,768 patients, whereas with the NAP5 estimate of approximately 1 in 19,600 about one event would be expected (3, 7). The absence of any reported event is compatible either with a genuine absence of effect or with the trial's inability to detect an effect of the magnitude suggested by earlier literature, and the trial was powered for postoperative delirium (26). B-Free therefore provides no reassurance on the awareness question. What it does show is that a BZD-free policy was not accompanied by an apparent increase in reported awareness, and that routine use of BZDs for this purpose can no longer be assumed to be necessary. A small or infrequent effect of BZDs on awareness in either direction remains possible.

The B-Free trial also examined postoperative outcomes. In the intention-to-treat analysis the primary result was neutral for delirium (14.0% with the restrictive policy vs. 14.9% with the liberal policy), with an adjusted odds ratio (aOR) of 0.92 and a 95% CI of 0.84 to 1.01 (*P* = 0.07) (26). In the per-protocol analysis, which excluded patients who did not receive the assigned policy (approximately 9.1% crossover in the restrictive arm), the restrictive policy was associated with lower odds of delirium (aOR 0.90, 95% CI 0.82 to 0.99, *P* = 0.02). We treat this as exploratory. It is not a confirmatory result. The upper limit of the confidence interval approaches unity, and per-protocol analyses are vulnerable to selection and adherence-related bias. Attributing the difference between the two analyses to dilution of the treatment effect by protocol nonadherence is plausible, but it is a hypothesis rather than a trial finding and cannot be tested with the data as presented. The delirium result should be regarded as hypothesis-generating and as requiring confirmation in adequately powered trials. Two further lines of evidence bear on the same question. A 2025 systematic review of 123 randomized controlled trials (*n* = 12,921), of which 93 were included in the quantitative synthesis, found that perioperative BZDs did not improve patient-reported outcomes and may increase postoperative anxiety; this review assessed patient-reported outcomes and did not evaluate delirium as an outcome (12). A prospective multicenter cohort study in patients older than 65 years reported an association between intraoperative midazolam and postoperative delirium (13). Because the design was observational, the association does not establish causation, and residual confounding by indication and by baseline risk of delirium remains possible (13, 23).

Remimazolam, an ultra-short-acting BZD, raises the question of whether newer agents alter this picture. The relevant evidence is indirect. A randomized non-inferiority trial in day-case surgery (*n* = 100) found no awareness events in either the remimazolam or the propofol group, with equivalent anesthetic success, better hemodynamic stability (32% vs. 68% requiring vasopressors) and less injection-site pain (4% vs. 80%) (28). A meta-analysis of 19 randomized controlled trials (*n* = 3,031) found no significant difference in awareness risk between remimazolam and propofol (RR 0.33, 95% CI 0.01 to 8.16, *P* = 0.502) alongside less intraoperative hypotension with remimazolam (RR 0.65, 95% CI 0.46 to 0.92) (29). Awareness was a secondary outcome in that analysis, and the confidence interval is compatible with both substantial benefit and substantial harm. The same authors noted a possible increase in delirium with remimazolam among patients of ASA physical status III–IV, a signal requiring prospective confirmation (29). A separate meta-analysis of 119 randomized controlled trials found that perioperative BZDs reduce postoperative nausea and vomiting, indicating that some of the benefits attributed to BZD premedication lie outside the domain of awareness (22). A GRADE-assessed meta-analysis of nine studies (*n* = 903) in adult cardiac surgery reported fewer intraoperative hypotensive events (RR 0.50; 95% CI 0.31–0.80) and faster emergence with remimazolam than with propofol (30). Two limitations apply to this body of work. It addresses anesthetic efficacy, hemodynamic stability and recovery, not the prevention of awareness, and the use of remimazolam as a primary anesthetic falls outside our scope. These studies are therefore contextual only. They indicate that adequately titrated modern techniques can achieve an appropriate depth of anesthesia without BZDs; they do not show that BZDs lack value as adjunctive amnestic agents, which is the question at issue. Whether BZDs add such value when depth is otherwise assured remains unresolved on the available evidence.

### Clinical implications: from pharmacologic amnesia to depth-centered management

Translating this evidence into practice calls for a structured approach. The recommendations below fall into three groups, and we identify each: recommendations derived from professional guidelines, recommendations supported by specific prospective studies, and propositions that represent our own judgment where direct evidence is limited. Preoperative identification of patients at increased risk of awareness is essential. The risk profile is well established and includes female sex, younger age, obesity, a history of awareness, chronic use of benzodiazepines, opioids or alcohol, and procedures performed with neuromuscular blockade (7). Existing advisories recommend that such patients be considered for an awareness-prevention strategy, including consideration of BZD administration (15). Patients at high risk of postoperative delirium should also be identified, in whom the balance between prevention of awareness and avoidance of delirium must be weighed individually. This last point is our own proposal rather than a guideline recommendation. Intraoperative monitoring should extend beyond traditional vital signs, a recommendation supported by specific studies. Heart rate and blood pressure are unreliable indicators of anesthetic depth, particularly in patients receiving *β*-blockers or in hemodynamically unstable conditions, and neuromuscular blockade removes the motor signs that might otherwise alert the practitioner to insufficient depth (7, 15). For patients at substantially increased risk of awareness, and during total intravenous anesthesia, a processed electroencephalographic index such as the bispectral index (BIS) is a reasonable adjunct (15, 31, 32). The evidence for its superiority over an end-tidal concentration-guided protocol is limited. In two randomized comparative-effectiveness trials in patients at high risk of awareness, the B-Unaware trial (Avidan and colleagues, 2008) and the BAG-RECALL trial (Avidan and colleagues, 2011), BIS-guided management did not reduce the incidence of awareness compared with a protocol based on end-tidal anesthetic gas concentration (33, 34). This is consistent with the conclusions of the two Cochrane reviews that examined depth-of-anesthesia monitoring. The review of bispectral index monitoring by Lewis and colleagues reported that BIS-guided anesthesia was associated with a lower frequency of intraoperative awareness than monitoring based on clinical signs (Peto odds ratio 0.36, 95% CI 0.21 to 0.60; 27 studies, 9,765 participants), and no evidence of a difference compared with monitoring based on end-tidal anesthetic gas concentration (Peto odds ratio 1.13, 95% CI 0.56 to 2.26; 5 studies, 26,572 participants), but the authors graded all of these estimates as low-certainty evidence, because events were rare and the trials were too imprecise to exclude clinically important differences (35). The broader review of anesthetic interventions by Messina and colleagues reached a comparable conclusion for processed electroencephalographic monitoring in general (24). Processed electroencephalographic monitoring is therefore best understood as an adjunct to clinical judgment and protocolized care, not as a replacement for either. Anesthesia-specific protocols should address the most common failure points: verifying an adequate level of sedation before administering neuromuscular blocking agents, regularly checking infusion pump function and intravenous line integrity during total intravenous anesthesia, and maintaining end-tidal volatile concentrations appropriate to age during volatile-based anesthesia when hemodynamic tolerance permits. A threshold near 0.7 age-adjusted minimum alveolar concentration is commonly discussed as being associated with a low probability of recall; this value derives from prospective trials but was characterized in patients at high risk of awareness rather than established as a universal target (33, 34). Lower concentrations are sometimes unavoidable in hemodynamically unstable patients, in whom vigilance and adjunctive depth monitoring assume greater importance. Standardized, protocolized management of anesthetic depth has not been shown in large-scale implementation trials to reduce awareness in unselected surgical populations, and this remains a gap in the evidence supporting our proposal. These measures are neither novel nor complex. They represent the disciplined application of principles that should already form the backbone of anesthetic practice.

## Discussion

The evidence reviewed here supports a reassessment of how intraoperative awareness prevention is framed. The available data do not establish a reliable awareness-preventive benefit of routine BZD administration, and the same randomized evidence does not establish a clear increase in postoperative delirium. Observational and secondary analyses raise that possibility and warrant further study (12–14, 26). The alternative we propose is not complex: the disciplined application of fundamental anesthetic principles, with attention to the maintenance of adequate depth, the use of appropriate monitoring in selected patients, and vigilance during administration. This is a proposal for a shift in emphasis, not an assertion that perioperative BZDs should be abandoned. They retain indications unrelated to awareness prevention, and advisories continue to list them among the options for patients at high risk of awareness (15).

### Strengths and limitations

The principal strength of this article is that it assembles in one place the randomized and systematic evidence bearing on a widely held but rarely tested rationale, and that it separates what the evidence shows from what we infer. Several limitations qualify our conclusions. The article is a Perspective rather than a systematic review. The literature was selected and not exhaustively retrieved, so study selection was subject to our own judgment and may have introduced selection bias. Independence of the evidence base is limited. The two systematic reviews are related to one another and to the B-Free investigators, and the same group of trials and cohorts reappears across several of the sources cited here (12–14, 21–23, 26). The studies cited span different surgical populations, agents and dosing regimens, and they used heterogeneous methods of ascertaining awareness. In most studies awareness was not assessed by a structured interview, which biases estimates toward the null. Several of the trials and cohorts are small, and some are at high or unclear risk of bias. The delirium signal is derived mainly from secondary, subgroup and observational analyses, and causality cannot be inferred from it. The evidence on remimazolam is indirect, since it addresses anesthetic efficacy and recovery and not awareness prevention. The external validity of the strongest evidence is limited. B-Free was conducted exclusively in cardiac surgery, so its findings should not be extended to obstetric anesthesia, trauma resuscitation or other settings in which the conditions of the trial, including the feasibility of achieving a consistent depth of anesthesia, may not hold. Our recommendations regarding routine depth monitoring during total intravenous anesthesia and structured preoperative risk stratification are consistent with existing advisories but have not been tested in large-scale implementation trials, and the cost-effectiveness of universal processed electroencephalographic monitoring, particularly in resource-limited settings, remains unresolved. Finally, the certainty of the available evidence for most outcomes remains low or very low, and our conclusions should be read within these constraints.

Surgeons and anesthetists share responsibility for perioperative safety, and decisions about prophylactic BZD administration sit at the intersection of that shared responsibility. On the present evidence, awareness prevention does not provide a secure basis for the routine administration of BZDs, and the risks associated with that practice, including postoperative delirium in vulnerable patients, deserve weight in the decision (12, 13). The evidence does not permit the opposite conclusion either. Clinicians should continue to identify patients at increased risk, to ensure that an adequate depth of anesthesia is achieved and monitored, and to consider BZDs where the risk of awareness is judged to be high (15). Unconsciousness during anesthesia is a physiological state to be achieved and verified, and pharmacologic amnesia is not a substitute for that verification. Routine use of BZDs as a replacement for careful depth management warrants reassessment.

## Data Availability

The original contributions presented in the study are included in the article/Supplementary Material, further inquiries can be directed to the corresponding author.
